# Enhancing Effects of NMDA-Receptor Blockade on Extinction Learning and Related Brain Activation Are Modulated by BMI

**DOI:** 10.3389/fnbeh.2017.00034

**Published:** 2017-03-07

**Authors:** Anne Golisch, Stefanie Heba, Benjamin Glaubitz, Martin Tegenthoff, Silke Lissek

**Affiliations:** Department of Neurology, BG University Hospital Bergmannsheil, Ruhr-University BochumBochum, Germany

**Keywords:** extinction learning, renewal effect, memantine, BMI, hippocampus

## Abstract

A distributed network including prefrontal and hippocampal regions is involved in context-related extinction learning as well as in renewal. Renewal describes the recovery of an extinguished response if the context of extinction differs from the context of recall. Animal studies have demonstrated that prefrontal, but not hippocampal *N*-methyl-*D*-aspartate receptor (NMDAR) antagonism disrupted extinction learning and processing of task context. However, human studies of NMDAR in extinction learning are lacking, while NMDAR antagonism yielded contradictory results in other learning tasks. This fMRI study investigated the role of NMDAR for human behavioral and brain activation correlates of extinction and renewal. Healthy volunteers received a single dose of the NMDAR antagonist memantine prior to extinction of previously acquired stimulus-outcome associations presented in either identical or novel contexts. We observed better, and partly faster, extinction learning in participants receiving the NMDAR antagonist compared to placebo. However, memantine did not affect renewal. In both extinction and recall, the memantine group showed a deactivation in extinction-related brain regions, particularly in the prefrontal cortex, while hippocampal activity was increased. This higher hippocampal activation was in turn associated with the participants' body mass index (BMI) and extinction errors. Our results demonstrate potentially dose-related enhancing effects of memantine and highlight involvement of hippocampal NMDAR in context-related extinction learning.

## Introduction

Extinction is an important learning phenomenon in everyday life, which allows organisms to adapt their behavior to new situations. This adaption involves new inhibitive or integrative learning rather than unlearning (Bouton, 2002; Phelps et al., 2004): Instead of erasing the established memory trace (Bouton, 2002; Quirk and Mueller, 2008), extinction learning presumably forms a new memory trace that competes with the initially acquired trace. Selection of a proper response thus involves choosing the adequate memory trace, a process for which context consideration may be crucial (for review see Rosas et al., 2013). The context-dependency of extinction is impressively illustrated by the renewal effect, which describes the recovery of an extinguished response when the test context differs from the extinction context (Bouton and Bolles, 1979). The most stable renewal effect is the so-called ABA renewal, in which an association between a conditioned (CS) and unconditioned stimulus (US) in context A is extinguished by repeating the CS without the US in context B, but recovered when the CS is presented again in context A (Harris et al., 2000; Bouton, 2004). The occurrence of the renewal effect illustrates that the context is integrated particularly in ambiguous situations (Rosas and Callejas-Aguilera, 2006).

Hippocampus, as a context processing brain area (Corcoran et al., 2005; Ji and Maren, 2005), as well as prefrontal (Quirk et al., 2000) and amygdalar regions (Davis et al., 2003) play a crucial role in extinction learning and the renewal effect, as demonstrated in animal studies (for a review see Maren et al., 2013). Recent functional magnetic resonance imaging (fMRI) studies were able to identify participation of these regions also in humans: The human ventromedial prefrontal cortex (vmPFC) and the hippocampus exhibit context-dependent responding, indicated by higher activation in the extinction context in contrast to the acquisition context (Phelps et al., 2004; Kalisch et al., 2006). Furthermore, a positive correlation was found between vmPFC and hippocampal activation during extinction recall (Milad et al., 2007). This network of brain areas mediating the recall of extinction memory is crucially involved in the occurrence of the renewal effect (Lissek et al., 2013).

In view of their high density in these extinction-related areas (Böckers et al., 1994; Wang and Arnsten, 2015), *N*-methyl-*D*-aspartate receptors (NMDAR) can be considered as a candidate for modulating extinction learning and renewal (for review see Myers and Davis, 2002). Indeed, animal studies showed that NMDAR antagonism in prefrontal regions impaired instrumental extinction learning (Lissek and Güntürkün, 2003; Quirk and Mueller, 2008) as well as reversal learning (Lissek et al., 2002; Bohn et al., 2003) and processing of task context (Lissek and Güntürkün, 2005). However, another study found no impairment of fear extinction after local medial prefrontal cortex (mPFC) NMDAR blockade (Laurent and Westbrook, 2008). In addition, systemic blockade of NMDAR as well as local infusions of NMDAR antagonists into the vmPFC caused deficits in the consolidation of extinction (Baker and Azorlosa, 1996; Santini et al., 2001; Burgos-Robles et al., 2007; Sotres-Bayon et al., 2009; for review see Davis, 2011). Overall, these findings suggest that NMDAR in prefrontal regions appear to play a role in extinction learning with or without a fear component.

In contrast, the role of hippocampal NMDAR for fear conditioning yielded divergent results. While NMDAR blockades in the hippocampus disrupted the acquisition of contextual fear conditioning (Quinn et al., 2005; Schenberg and Oliveira, 2008), involvement of hippocampal NMDAR in fear recall appears contradictory: Studies found (Fiorenza et al., 2012) or did not find (Quinn et al., 2005; Chang and Liang, 2012) detrimental effects of NMDAR antagonists upon the retrieval of contextual fear memory. In contrast, hippocampal NMDAR antagonism did not impair context processing in appetitive (Good and Bannerman, 1997) and fear conditioning (Tayler et al., 2011). Overall, animal studies provide evidence for potential effects of NMDAR antagonists upon context-related extinction learning. However, studies in humans are still lacking.

In the present study, we examined the role of an NMDAR antagonist on contextual extinction learning and the renewal effect in healthy human participants. We used the non-competitive NMDAR antagonist memantine, which has previously been shown to impair learning processes in humans, such as object recognition (Rammsayer, 2001) as well as tactile perceptual learning (Dinse et al., 2003) and classical eyeblink conditioning (Schugens et al., 1997). Additionally, a single dose of memantine showed the potential to decrease brain activation in fronto-striatal-parietal networks (Jamadar et al., 2012). Memantine is frequently used in treatment of neurodegenerative disorders, with the aim of enhancing the cognitive performance of patients, e.g. in memory and language (for review see Parsons et al., 2007; Lanctot et al., 2009). Due to effects observed in patients, memantine was recently discussed as a potential neuroenhancer for healthy humans (for review see Repantis et al., 2010). However, up to now only a few studies determined enhancing effects of a single dose of memantine in healthy human participants, reporting for instance an increase in processing speed (Korostenskaja et al., 2007). Yet, research on acute memantine effects upon learning is still rare. Moreover, potential dose-dependent effects have not yet been investigated in healthy humans. Animal studies usually determine dosage based on body weight and already suggested dose-dependent effects of NMDAR antagonists (e.g., AP5/MK-801) revealing impaired extinction learning under high doses (Falls et al., 1992) and conversely enhanced performance under low doses (Baker and Azorlosa, 1996). In contrast, in healthy humans corresponding literature is still lacking, and most studies use a standardized single dose irrespective of the participants' body weight or body mass index (BMI).

To investigate the neuronal correlates of extinction and renewal outside a fear context, we used an associative learning task in which participants were required to learn relations between cues and outcomes presented in particular contexts. This predictive learning task (Ungör and Lachnit, 2006), which we already used in previous studies (Lissek et al., 2013, 2015a,b), features an ABA design previously shown to evoke a renewal effect. In contrast to fear conditioning, no aversive stimuli respectively consequences were used. Despite the differences in task properties, this associative learning task, however, has previously shown to evoke brain activation patterns (Lissek et al., 2013, 2015a,b, 2016) similar to fear extinction paradigms (in humans and animals; for review see Sehlmeyer et al., 2009).

In the present study, healthy volunteers received a single dose of the NMDAR antagonist memantine (MEM group) or a placebo (PLAC group) prior to extinction of previously acquired stimulus-outcome associations presented in either identical (AAA condition) or novel (ABA condition) contexts. We assumed that blocking activation in the NMDA system would lead to impaired extinction learning performance compared to the placebo group. In consequence, we expected a more prominent renewal effect in participants receiving the NMDAR antagonist. In accordance, we hypothesized that brain areas which are involved in extinction learning and have a high density of NMDAR, such as prefrontal and hippocampal regions, would show a decreased activation level compared to the placebo group.

## Materials and methods

### Participants

A group of 64 healthy participants (34 males, 30 females) volunteered in this study. Six subjects (PLAC *N* = 4; MEM *N* = 2) had to be excluded due to weak learning performance (i.e., overall percentage of correct responses during acquisition <70%). Additionally, 10 subjects were excluded due to medical side effects like nausea and vomiting (MEM *N* = 3), or inadequate datasets, including neurological abnormalities (PLAC *N* = 1) and signal or movements artifacts (PLAC *N* = 5; MEM *N* = 1). All reported analyses are calculated from the final sample of 48 participants with 24 subjects per group (24 males, 24 females; mean age 25.00 ± 0.55 years SEM, range 19–38 years). All participants had normal or corrected-to-normal vision; none had any current neurological and medical condition. Only right-handed participants (Edinburgh Handedness Inventory; Oldfield, 1971) were recruited (mean quotient 81.68 ± 2.94). Participants received a monetary compensation (in the amount of 60€) and were randomly allocated to the experimental memantine (MEM) or control placebo (PLAC) group. Both groups included equal proportions of men and women (PLAC χ^2^ = 0.000, *p* = 1.00; MEM χ^2^ = 0.000, *p* = 1.00) and did not differ significantly in age [*t*_(46)_ = 0.755, *p* = 0.454; PLAC mean age 25.58 ± 0.78 years SEM, MEM mean age 25.42 ± 0.78]. For fMRI measurements participants' body weight and height were recorded and the resultant BMI was calculated for each subject. The groups did not differ significantly in their BMI [*t*_(46)_ = 1.545, *p* = 0.129; PLAC 25.17 ± 0.83, MEM 23.45 ± 0.76] (BMI data acquired via self-reported questionnaires).

Subjects were recruited via local advertisements and participated after giving written informed consent. This study was approved by the local ethics board of the Ruhr University Bochum and was carried out in accordance with the Ethics of the Word Medical Association (Declaration of Helsinki). Prior to the experiment, participants read written instructions informing them about the pharmacological properties of the NMDAR antagonist memantine, its general clinical use and the experimental fMRI procedure.

### Predictive learning task

The predictive learning tasked was originally conceived by Ungör and Lachnit (2006) in order to investigate context-dependency of associative learning and the renewal effect. Its efficiency was demonstrated in several behavioral as well as fMRI studies, which investigated context-dependent extinction learning (Lissek et al., 2013, 2015a,b). Moreover, this study design has previously been adapted to examine variations in renewal (ABA, ABC, AAB) and their manipulations (e.g., context relevance) (Üngör and Lachnit, 2008; Lucke et al., 2014) as well as the effects of pharmacological modulations on extinction and retrieval (e.g., cortisol, dopamine, noradrenalin) (Hamacher-Dang et al., 2013; Lissek et al., 2015a,b).

In the predictive learning task, participants are asked to put themselves in the situation of a physician and predict whether their patient will suffer from stomachache after consuming diverse food items served in different restaurants. In this way, participants learn to associate several stimuli (food items) with particular consequences (occurrence or non-occurrence of a stomachache) in different contexts (restaurants).

**Figure 1:**
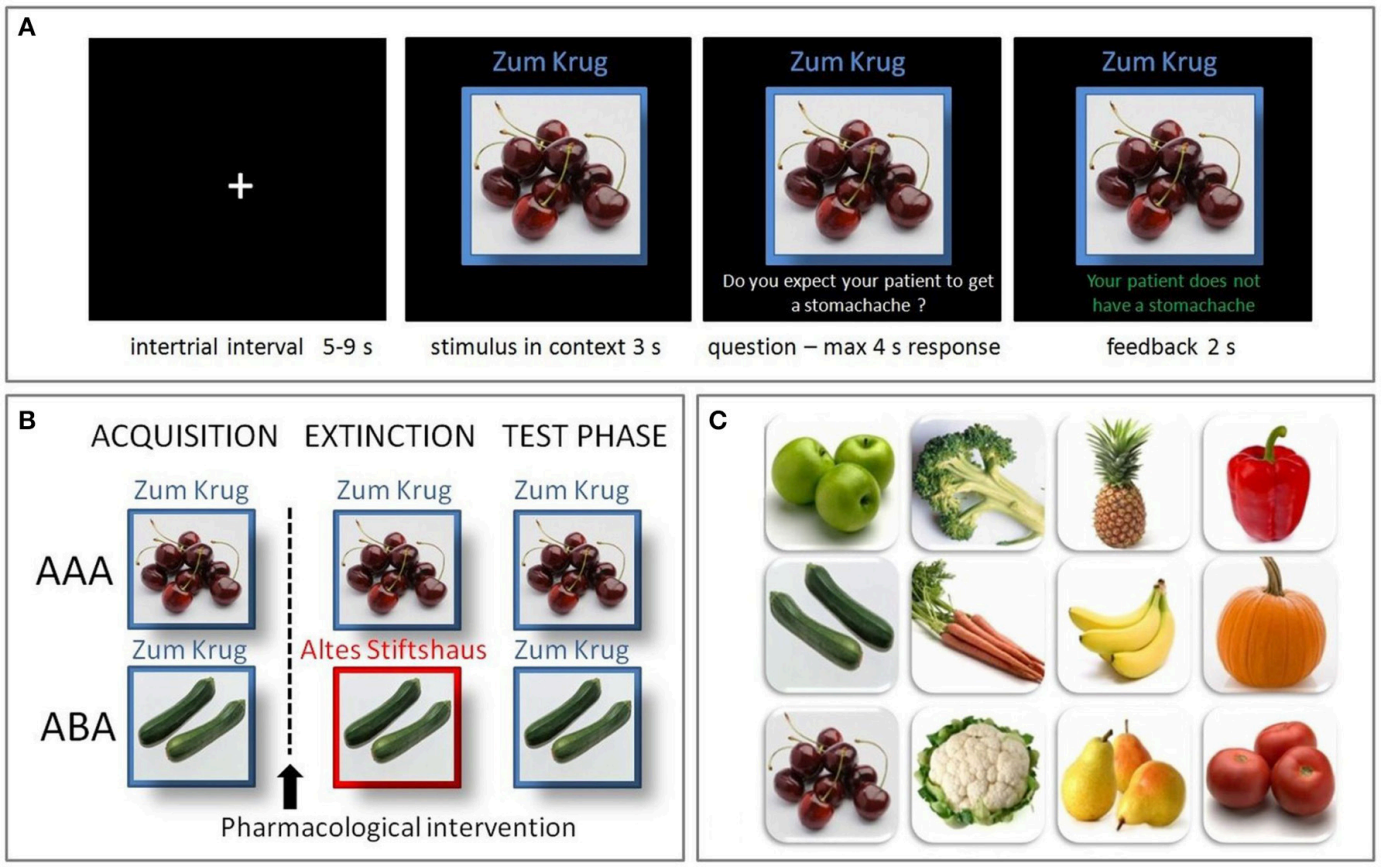
**Predictive learning task**. **(A)** Example trial of the predictive learning task. Participants learned the association between a food item, eaten in a specific restaurant, and a specific consequence. After an intertrial interval of 5–9 s the stimulus and its context was presented for 3 s. Then a question was superimposed on the screen, asking whether the patient will suffer from stomachache, followed by a response period of max. 4 s. A feedback, providing the correct answer, was presented for 2 s. **(B)** The predictive learning task consists of three learning phases: acquisition, extinction and recall. In the AAA condition, all phases occur in the same context, while in the ABA condition the extinction context differs. In both conditions, in the recall phase stimuli are presented in the same context as during acquisition. **(C)** Food images used in the task.

The learning process was divided into three phases: Acquisition, extinction, and recall. During the acquisition phase participants learned the association between a food item and a specific consequence. In each trial, 1 of 12 food stimuli (fruit or vegetable; see Figure 1C) was presented in one of two contexts. The context was indicated by the name of the restaurant (“Zum Krug/The Mug” or “Altes Stiftshaus/The Dome”) and a frame in either red or blue color. The stimulus-context combination was presented for 3 s. Then a question asking whether the patient will suffer from stomachache was presented on the screen. Participants had to respond with yes or no using the assigned keys on a fMRI-ready keyboard (Lumitouch response pad, Photon Control Inc., Canada) within 4 s. Immediately after their response, or in case of a missing response at the end of the response period, the feedback with the correct answer was presented for 2 s: “The patient has a stomachache” (written in red color) or “The patient does not have a stomachache” (written in green color; see Figure 1A). Six stimuli appeared per context. Each stimulus-context-feedback combination was presented eight times, adding up to a total of 96 single trials. Stimulus order and assignment to contexts and consequences was randomized across sessions.

In the extinction phase (96 trials), half of the stimuli were presented in the same context as during acquisition (AAA condition, 48 trials), while for the other half of the stimuli the context changed (ABA condition, 48 trials) (see Figure 1B). Within these conditions, stimuli were subdivided into two types: First, the actual extinction trials, where the consequence of stomach trouble changed and the new consequence had to be learned and second, the distractor trials, where the consequence did not change. The latter were introduced in order to make the overall learning more complex and therefore difficult. In each context a consequence change was present in four stimuli (AAA ext/ABA ext), while it was absent in two stimuli (AAA dist/ABA dist). All other aspects of the acquisition task remained the same in the extinction phase.

During the recall phase (60 trials), all stimuli were again presented in the context of acquisition (five presentations), whereupon no feedback was given. No further aspects were changed compared to the acquisition phase.

### Procedure

Two MR sessions comprising structural and functional imaging were conducted on 2 consecutive days in order to enable consolidation of acquisition. On day 1, first a structural T1-weighed image was recorded, after which participants completed the acquisition phase of the predictive learning task in a first fMRI session.

Twenty-four hours later, on day 2, half of the participants received a single oral dose of 30 mg memantine, while the other half received an identical-looking placebo and acted as a control group. The 30 mg dose of memantine was previously used in other several studies on learning and memory and yielded significant effects e.g., on object recognition and eyeblink conditioning (Schugens et al., 1997; Rammsayer, 2001, 2003, 2006; Korostenskaja et al., 2007; Swerdlow et al., 2009).

After drug administration, participants rested for 2.5–3 hrs, after which the second fMRI session, comprising extinction learning and recall phases, was performed. The timing was based on the pharmacological profile of memantine and its time to peak (Schwenkreis et al., 2005).

#### Imaging data acquisition

Structural and functional imaging was performed on a whole-body 3.0 Tesla scanner (Philips Achieva 3.0 T X-Series, Philips, The Netherlands) with a 32-channel SENSE head coil. High resolution structural brain images, using an isotropic T1-weighted TFE sequence (TR 8.2 ms, TE 3.7 ms, field of view 240 mm, slice thickness 1 mm, 220 transversal slices with a voxel size of 1 × 1 × 1 mm^3^), were acquired for each participant. For the functional analysis, blood-oxygen level dependent (BOLD) contrast images were acquired with a dynamic T2^*^-weighted EPI-sequence (TR 3200 ms, TE 35 ms, flip angle 90°, field of view 224 mm, slice thickness 3 mm, 45 transaxial slices parallel to the anterior commissure-posterior commissure (AC-PC) plane with a voxel size of 2 × 2 × 3 mm^3^).

The task was presented to participants via fMRI-ready LCD-goggles (VisuaStim Digital, Resonance Technology Inc., Northridge, CA, USA) connected to a laptop that supported a specific software programmed in Matlab. Responses were reported via an fMRI-ready keyboard (Lumitouch response pad, Photon Control Inc., Canada).

#### Imaging data analysis

For preprocessing and statistical analysis of fMRI data we used the software Statistical Parametric Mapping (SPM), Version 8 (Wellcome Department of Cognitive Neurology, London, UK), implemented in Matlab R2008a (The Mathworks, Natick, MA, USA). Three dummy scans, during which the BOLD signal reached steady state, preceded the actual data acquisition of each session, thus preprocessing started with the first acquired volume. Preprocessing on single subject level consisted of the following steps: Slice timing correction to account for time differences due to multislice image acquisition; realignment of all volumes to the first volume for motion correction; spatial normalization into standard stereotactic coordinates with 2 × 2 × 2 mm^3^ using an EPI template of the Montreal Neurological Institute (MNI), smoothing with a 6 mm full-width half-maximum (FWHM) kernel, in accordance with the standard SPM procedure. The acceptable limit for head motion was 2 mm for translational movements and 0.5° for rotational movements.

In a first level single subject analysis, we calculated activation during extinction and recall phases in the conditions ABA and AAA, respectively. The contrasts were calculated within a combined anatomically defined mask which was constructed using the software MARINA (BION Bender Institute of Neuroimaging, University of Giessen, Germany) (Walter et al., 2003). The mask consisted of literature-based *a priori* regions of interest, which have previously been shown to constitute parts of the network highly involved in the extinction (for review see Sehlmeyer et al., 2009), containing bilateral prefrontal cortex (OFC, dlPFC, vmPFC, ACC), hippocampus, amygdala, insula, and temporal lobe. All data contained in this combined mask were analyzed together in a single analysis. We used an event-related design, modeling the events of each trial (AAA and ABA ext, all ext and all dist), i.e., onsets of stimulus, question and feedback presentation, as stick functions convolved with the SPM default hemodynamic response function (HRF) in SPM. Our analyses were based on the stimulus presentation phase of each trial. For the contrasts of the extinction learning phase, only those stimuli were used for which the consequence of stomach trouble, learned during acquisition, changed during the extinction phase (i.e., ext stimuli). These contrast images were entered into second-level random-effects analyses. In one-sample tests we analyzed the activation patterns of the experimental and control groups for the different contrasts, using a threshold of *p* < 0.05 FWE-corrected (Family-Wise Error) on cluster level with a minimal cluster size (k) of 10 voxels (*k* = 10), adding age and gender as nuisance variables. Moreover, we calculated two-sample tests to directly investigate in which regions the experimental group showed differential activation compared to controls. To identify subtle group differences in extinction-relevant regions in a hypothesis-led anatomically constrained manner, we used a more liberal threshold of *p* < 0.001 uncorrected.

*Post-hoc*, we examined possible differential effects of memantine related to the participants' body weight in order to discover potential dose-dependent effects of memantine. Considering the lipophilic pharmacological characteristics of memantine (Henkel et al., 1982), we used participants' BMI as a covariate, which—by relating body weight to height—gives a better indication of their body stature, taking into account relative fat content and metabolism. Moreover, BMI strongly correlates with the body fat percentage (Ranasinghe et al., 2013), however, this association is influenced by age and gender (Gallagher et al., 2000; Jackson et al., 2002; Ranasinghe et al., 2013). Therefore, in addition to regressors modeling group and condition, we entered participants' BMI and percentage of extinction errors as covariates of interests into the analysis, using a threshold of *p* < 0.05 FWE-corrected on cluster level (*k* = 20) and age and gender as nuisance variables. For all reported analyses peak coordinates (MNI) and related *t*- and *p*-values as well as cluster sizes were reported in corresponding tables (see Tables 1–**3**).

**Table 1:** **One-sample tests (age and gender as nuisance variables)—activated regions (MNI coordinates) in MEM and PLAC during extinction learning, peak cluster *p* < 0.05 FWE-corrected, *k* = 10**.

**Brain region**	**BA**	**Hem**	**AAA CC**										**ABA CC**									
			**MEM**					**PLAC**					**MEM**					**PLAC**				
			**voxel**	**t-value**	***x***	***y***	***z***	**voxel**	**t-value**	***x***	***y***	***z***	**voxel**	**t-value**	***x***	***y***	***z***	**voxel**	**t-value**	***x***	***y***	***z***
dlPFC		R											20	6.04	44	46	24					
	8	R						141	6.04	48	16	48						153	6.96	50	20	44
		R						14	6.01	4	36	54										
		L						35	6.65	−50	10	46						46	6.09	−46	10	44
	9	R						187	8.53	54	20	38						161	6.3	44	34	36
		L						45	5.34	−48	20	40						23	5.41	−54	14	36
	45	R						14	5.44	58	24	26	12	5.92	60	22	20					
	46	R	13	5.92	46	44	20	105	6.97	46	42	22						98	7.26	46	42	22
		L																10	6.1	−54	24	26
OFC	10	R	38	6.3	44	48	16	225	8.87	34	58	22										
		L						36	5.22	−36	44	30						27	6.42	−40	50	16
	47	R	16	6.88	52	20	−10	53	5.89	56	26	−2						46	6.79	56	20	−4
		L	13	5.56	−50	18	−6	49	6.78	−46	18	−6						37	6.25	−46	18	−8
STG	38	R	29	5.68	50	20	−14	50	9.29	52	18	−12						62	9.74	52	18	−12
		L	26	6.48	−48	14	−8	38	6.95	−50	18	−10	13	5.38	−48	16	−8	56	6.54	−44	18	−20
	22	R						15	7.65	54	14	−6						12	6.53	54	14	−4
		L	17	7.37	−54	12	−6	21	8.18	−54	12	−6						20	8.29	−54	12	−6
Anterior cingulate	25	L	13	6.36	−6	20	−2															
Cingulate Gyrus		R						70	5.61	2	32	30						115	5.99	2	18	40
	32	R																47	6.3	4	26	36
Insula		R																16	5.87	48	16	−6
		L						18	5.57	−44	14	−10										
Amygdala		R						35	5.53	28	0	−12						26	6.49	26	0	−16
		L						11	5.02	−24	−2	−14										
Thalamus		R	18	5.62	22	−30	0	65	5.47	18	−30	0						78	7.12	22	−30	4
		L						14	5.87	−24	−32	4						12	6.24	−24	−32	4
Hippocampus		R	11	5.84	18	−32	−6	23	5.49	20	−32	−2						54	6.57	20	−30	−4
		L	22	5.56	−36	−18	−14						15	6.45	−16	−32	−6					
		L	24	6.54	−16	−30	−8															
Parahippocampal Gyrus		R	45	5.29	12	−46	−6											64	5.1	28	−30	−12
		L	27	6.04	−18	−32	−8	13	5.63	−26	0	−14										
	27	R											36	5.87	12	−36	−2	18	5.89	20	−32	−8
		L											20	5.58	−18	−32	−8	14	6.44	−22	2	−14
Lingual Gyrus		R	73	5.39	14	−36	−6	48	6.77	12	−32	−4	72	6.33	10	−38	−2	42	7.01	16	−32	−6
		L	14	6.18	−14	−32	−6	24	5.43	−8	−36	−2	19	5.01	−10	−34	−6					
	30	R	17	5.26	16	−36	−6															
	19	L																22	6.37	−14	−50	−2

#### Behavioral data analysis

Log files for all three learning phases were recorded, which contained information on response latency, response type and correctness of response, from which we calculated error rates. Errors in acquisition and extinction learning were defined as responses stating the incorrect association between the context–cue compound and the consequence. For calculation of the renewal effect, during the recall phase only responses to stimuli with a consequence change were analyzed. The behavioral renewal effect in the predictive learning task should occur only in the condition ABA, in which extinction is performed in a context different from the context present during acquisition and recall phase. During recall, a renewal effect occurs if a response is given that was correct during acquisition, but wrong during extinction (e.g., if during acquisition cherries in context A cause stomachache, and during extinction cherries in context B do not cause stomachache any more, then a renewal effect response during recall states that cherries in context A cause stomachache). Statistical analyses (*t*-tests, ANOVA, χ^2^ and correlations) were performed using the IBM SPSS Statistics for Windows software package, version 22.0 (IBM Corp, Armonk, NY, USA). To test our hypotheses, we used two-tailed *t*-tests. Only for tests of directional hypotheses we used one-tailed analyses. These particular tests are marked. In case of a necessary Greenhouse-Geisser correction, the ε value is added to the corresponding ANOVA results. For correlations, Spearman's correlation coefficient (*rho*) is reported in order to achieve results unaffected by outliers. All results are quoted as mean ± SEM, unless stated otherwise.

## Results

### Behavioral data

#### Acquisition

During acquisition the complete group of included participants achieved an average of 84.33% (± 1.08) correct responses overall. We observed no significant differences in acquisition performance between the PLAC and the (pre-treatment) MEM group: *t*_(46)_ = −1.468, *p* = 0.149 (percent errors mean: PLAC 17.23% ± 1.49; MEM 14.11% ± 1.53). The reaction times in acquisition learning did not differ significantly between the groups [*t*_(46)_ = 0.252, *p* = 0.802; PLAC 720 ms ± 40; MEM 740 ms ± 50].

#### Extinction

##### Overall extinction learning

**Figure 2:**
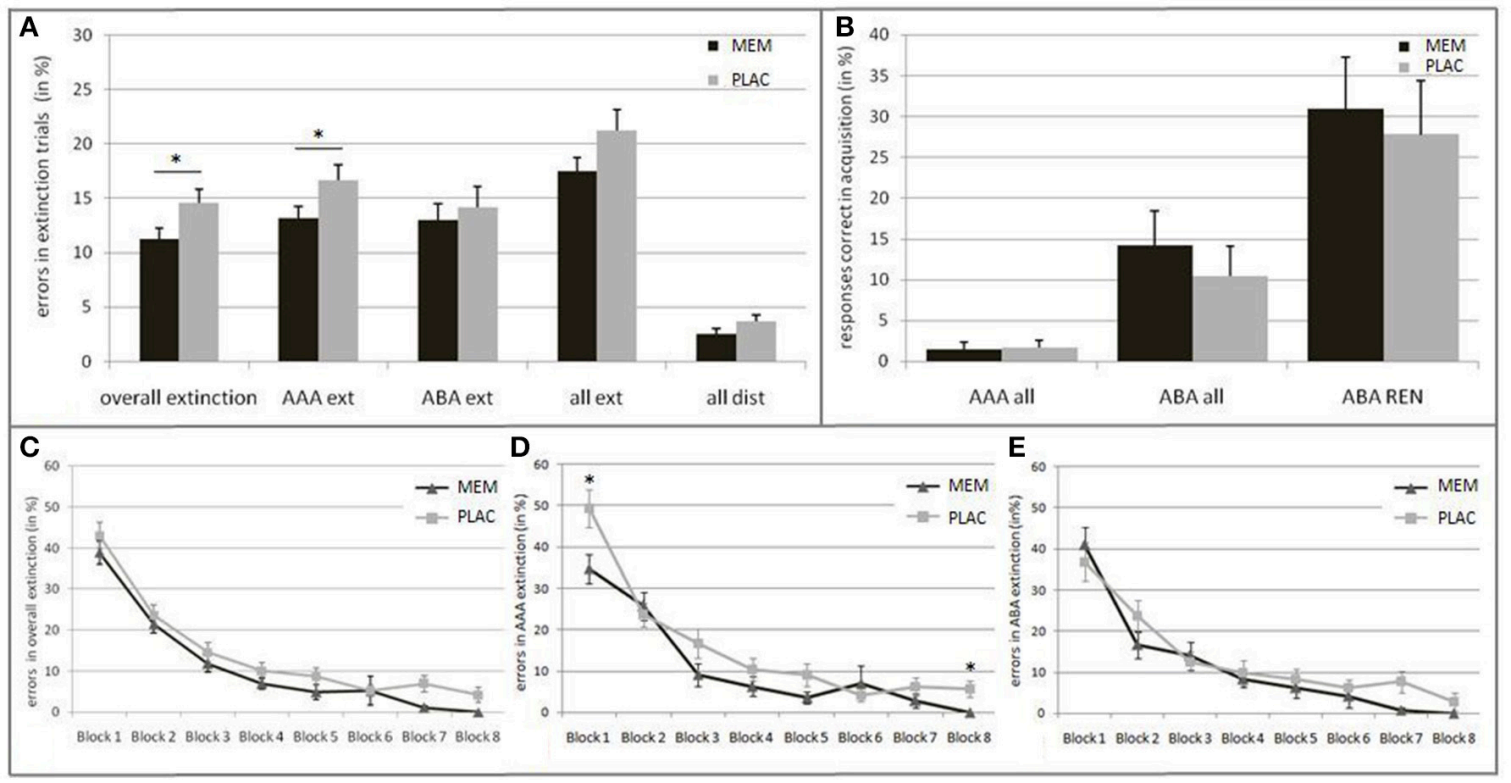
**Behavioral results in Extinction and Recall. (A)** Mean percentage of errors during extinction trials in the PLAC (gray) and MEM group (black). The MEM group made significantly fewer errors in overall extinction learning as well as in AAA extinction (AAA ext). No group differences were present in ABA extinction (ABA ext) and distractor trials (all dist). **(B)** Mean percentage of responses that were correct in acquisition during recall in condition AAA (i.e., errors) and ABA (i.e., renewal) in all participants, and only in those who showed the renewal effect (ABA REN); PLAC (gray), MEM (black). The groups did not differ in the strength of the renewal effect. **(C)** Extinction learning curves for the PLAC (gray) and MEM group (black) with the extinction session divided into eight blocks of 12 trials each. Learning progress is depicted for **(C)** overall extinction as well as for **(D)** condition AAA and **(E)** ABA. The MEM group showed significantly faster overall extinction learning progress that was based on better AAA extinction learning. No group differences were observed in ABA extinction learning progress. Error bars denote standard errors. ^*^p < 0.05.

Against our initial hypothesis, we observed no extinction learning impairment but rather an enhancement in the MEM group: Overall, the MEM group made significantly less errors than the PLAC group [*t*_(46)_ = −2.051, *p* = 0.046; PLAC 14.54% ± 1.29; MEM 11.29% ±.93]. To further examine the group differences we evaluated the learning progress over time. Thereto, the extinction session was subdivided into eight blocks, 12 trials each. For each block the percentage of errors was calculated. The repeated measures ANOVA showed a significant main effect of the repeated factor learning block [*F*_(7, 322)_ = 87.237, *p* = 0.000; ε = 0.640], as well as a significant main effect of group [*F*_(1, 46)_ = 4.204, *p* = 0.046]. In both groups, error rates declined across blocks, with no significant interaction [*F*_(7, 322)_ = 0.387, *p* = 0.910; ε = 0.640]. (See Figure 2C).

Importantly, it should be noted that early and late extinction learning trials seem to reflect different learning respectively memory processes: Whereas early trials typically reflect the persistence of the recently acquired, short-term conditioning memory, late trials better reflect extinction learning (Milad et al., 2007; Norrholm et al., 2015). Against this background, we grouped the extinction session into different phases: Initial examination of the extinction stimuli (1st block), early extinction learning (2nd–5th block) and late extinction learning phase (6–8th block). While MEM and PLAC had similar error rates during initial exposure to the changed stimulus-outcome contingencies [*t*_(46)_ = −0.970, *p* = 0.34; PLAC 43.06% ± 3.20; MEM 38.89% ± 2.87], during the following early extinction learning phase the MEM group tended to make fewer errors than PLAC [*t*_(46)_ = −1.735, *p* = 0.091; PLAC 14.58% ± 1.63; MEM 11.29% ± 0.99]. This performance difference increased during late extinction learning, where MEM participants showed significantly less errors [*t*_(46)_ = −2.531, *p* = 0.016; PLAC 5.44% ± 1.38; MEM 1.51% ± 0.71].

##### AAA and ABA extinction learning

To further explore this observed enhancement in MEM participants, we separately analyzed error rates for each trial category, i.e., AAA respectively ABA extinction trials (AAA ext/ABA ext), extinction trials in total (all ext) and distractor trials (all dist). *Post-hoc* tests showed significantly lower error rates in AAA ext trials for MEM participants compared to PLAC [*t*_(46)_ = −1.952, *p* = 0.029, one-tailed; PLAC 16.67% ± 1.44; MEM 13.15% ± 1.08]. In contrast, when considering extinction learning performance in a novel context (ABA ext) no significant group difference was present [*t*_(46)_ = −0.492, *p* = 0.313, one-tailed; PLAC 14.20% ± 1.84; MEM 13.02% ± 1.51]. Taken together, a trend toward a significant group difference can be observed regarding extinction errors in total (all ext): *t*_(46)_ = −1.648, *p* = 0.053, one-tailed; PLAC 21.27% ± 1.91; MEM 17.45% ± 1.32. In addition, a trend toward a better memory for associations in distractor trials (all dist) was observed in MEM compared to PLAC participants [*t*_(46)_ = −1.540, *p* = 0.065, one-tailed; PLAC 11.59% ± 1.76; MEM 7.95% ± 1.58]. (See Figure 2A).

To evaluate the condition-specific learning progress, we analyzed AAA and ABA extinction trials separately. The repeated measures ANOVA showed a significant main effect of the repeated factor learning block upon AAA error rates [*F*_(7, 322)_ = 50.537, *p* = 0.000; ε = 0.703] as well as a significant main effect of group [*F*_(1, 46)_ = 6.036, *p* = 0.018]. Despite the faster AAA extinction learning progress in the MEM group compared to PLAC, a significant interaction of AAA learning blocks × group [*F*_(7, 322)_ = 2.092, *p* = 0.044; ε = 0.703] can be reported. *Post-hoc* test showed a significant group difference in the initial [1st block: *t*_(46)_ = −2.479, *p* = 0.017; PLAC 49.31% ± 4.65; MEM 34.72% ± 3.61] and in the last extinction learning block [8th block: *t*_(46)_ = −2.892, *p* = 0.008; PLAC 5.56% ± 1.92; MEM 0.00% ± 0.00]. (See Figure 2D).

The ABA extinction learning progress was analyzed in the same way. A repeated measures ANOVA showed a significant main effect of the repeated factor learning block upon ABA error rates [*F*_(7, 322)_ = 38.478, *p* = 0.000; ε = 0.567]. However, we observed no significant effect for the factor group [*F*_(1, 46)_ = 1.266, *p* = 0.266] and for the interaction ABA learning blocks × group [*F*_(7, 322)_ = 0.919, *p* = 0.492; ε = 0.567], indicating that the PLAC and MEM group showed a comparable learning progress in ABA ext trials. (See Figure 2E).

Reaction times in extinction learning did not differ significantly between MEM and PLAC participants [*t*_(46)_ = −0.823, *p* = 0.415; PLAC 680 ms ± 50; MEM 630 ms ± 40].

#### Recall

As hypothesized, the complete group retrieved associations correct during acquisition (i.e., renewal) significantly more frequently in ABA recall, where extinction occurred in a different context, than in AAA recall, where all learning phases occurred in an identical context: *t*-test for matched samples *t*_(47)_ = −3.678, *p* = 0.001; ABA 12.29% ± 2.81; AAA 1.56% ± 0.63. However, blocking NMDAR did not significantly affect the ABA renewal level compared to the PLAC group: *t*_(46)_ = 0.663, *p* = 0.511; PLAC 10.42% ± 3.69; MEM 14.17% ± 4.30. Likewise, no significant differences between the PLAC and the MEM group were observed for errors in AAA recall trials [*t*_(46)_ = −0.163, *p* = 0.872; PLAC 1.67% ±.94; MEM 1.46% ± 0.88). (See Figure 2B).

Half of the participants (24 out of 48) did not show any renewal effect at all—these participants responded consistently according to the associations learned in the extinction phase. Participants who showed (REN) or did not show (NoREN) ABA renewal were equally distributed in the PLAC (χ^2^ = 1.500; *p* = .221; REN 37.5%; NoREN 62.5%) as well as in the MEM group (χ^2^ = 0.167; *p* = 0.683; REN 45.8%; NoREN 54.2%). Analyzing only the REN subgroups showed no significant influence of the NMDA antagonist memantine on the strength of ABA renewal compared to the untreated PLAC group [*t*_(18)_ = 0.338, *p* = 0.739; PLAC 27.78% ± 6.62; MEM 30.91% ± 6.39].

No significant differences were observed with regard to the reaction times in recall [*t*_(46)_ = −0.011, *p* = 0.991; PLAC 520 ms ± 40; MEM 520 ms ± 40].

### Imaging results

To determine brain activation patterns during extinction and recall phases in familiar (AAA) or novel contexts (ABA) in the PLAC and MEM groups, we analyzed the data separately for each group, including age and gender as nuisance variables.

#### Activation patterns of MEM and PLAC during extinction learning and recall

##### Extinction

During extinction learning in an identical context (AAA condition), both groups showed prominent activation in dorsolateral prefrontal cortex (dlPFC) [Brodmann Area (BA) 46] and orbitofrontal cortex (OFC) (BA10,47) as well as in superior temporal gyrus (STG) (BA22,38). In addition, we observed activation clusters in thalamus and lingual gyrus as well as in anterior cingulate respectively cingulate gyrus for both groups. PLAC participants in contrast to MEM, however, exhibited larger clusters and a more extensive activation particularly in frontal [OFC, dlPFC (BA8,9)] as well as in limbic structures (insula, amygdala). In contrast, hippo- and parahippocampal activations were more dominant in MEM participants compared to PLAC (see Table 1).

During extinction learning in a novel context (ABA condition), PLAC participants' activation patterns were similar to those shown in the familiar context, while MEM participants showed considerably decreased activation. Thus, in frontal and temporal areas the MEM group only activated a small cluster in dlPFC (BA45) and STG (BA38), while PLAC participants additionally exhibited activation of substantially larger clusters also in BA8, 9 and 46 as well as in OFC (BA10,47) and STG (BA38,22). Although, both groups showed activations in hippo- and parahippocampal brain regions (BA27) as well as in lingual gyrus (BA19), activation patterns in PLAC participants were more dominant and complex compared to MEM. Moreover, only PLAC participants exhibited activations in cingulate gyrus (BA32) and limbic system as well as in thalamus (see Table 1).

##### Recall

During extinction recall, where all stimuli were again presented in the context of acquisition, both groups showed similar activations in condition AAA and ABA. Both groups activated dlPFC (BA8,9), OFC (BA47), cingulate gyrus (BA24,32), and left transverse temporal gyrus (BA41). Additionally, both groups exhibited activation in insula, hippocampus and fusiform gyrus. MEM participants, compared to PLAC, showed higher activation in hippocampus and thalamus, whereas in frontal and insula regions a lower activation was observed (see Table 2).

**Table 2:** **One-sample tests (age and gender as nuisance variables)—activated regions (MNI coordinates) in MEM and PLAC during extinction recall, peak cluster *p* < 0.05 FWE-corrected, *k* = 10**.

**Brain region**	**BA**	**Hem**	**AAA CC**										**ABA CC**									
			**MEM**					**PLAC**					**MEM**					**PLAC**				
			**voxel**	***t*-value**	***x***	***y***	***z***	**voxel**	***t*-value**	***x***	***y***	***z***	**voxel**	***t*-value**	***x***	***y***	***z***	**voxel**	***t*-value**	***x***	***y***	***z***
dlPFC	8	R						21	5.62	4	18	48										
		L	21	6.19	−4	16	48						19	5.78	−2	18	48					
	9	R											13	5.84	48	6	38	12	5.4	52	8	38
		L						29	7.53	−56	10	36						14	6.76	−56	10	36
OFC	10	L											10	5.43	−34	44	28					
	47	R	16	6.26	52	20	−8	34	5.85	36	22	−6	25	5.54	50	18	−6	21	5.93	40	22	0
		L						22	5.15	−28	22	−8										
STG	38	L	11	5.33	−48	14	−10															
	22	R																25	5.73	54	6	0
		L	13	5.22	−48	0	2	22	5.4	−52	4	2	37	5.7	−50	4	−2	33	6.42	−48	0	0
Transverse temp. Gyrus		L																13	5.35	−48	−20	12
	41	L	10	6.26	−50	−20	10	15	5.91	−50	−18	10	13	7.46	−52	−18	12					
Cingulate Gyrus	32	R						211	6.95	8	22	38						101	6	4	16	42
		L	131	5.91	−2	4	52						167	6.3	−2	14	46					
	24	R						21	5.28	6	12	36	32	5.27	8	22	28	18	5.18	6	2	50
Insula		R	129	6.99	36	24	−6	191	7.16	36	24	−6	119	6.57	36	24	−6	124	6.51	36	24	0
																		85	6.36	40	2	4
		L	33	5.26	−42	2	2	193	7.44	−30	22	6	180	7.44	−32	18	8	122	7.13	−46	2	0
		L	18	5.86	−32	16	8	37	6.41	−46	0	2						16	5.54	−30	22	6
Insula	13	R	34	5.75	36	18	8	25	5.76	36	20	4	16	5.54	36	22	2	21	6.16	36	22	2
		R																30	5.59	42	6	4
		L						24	5.21	−36	20	4	54	5.11	−34	16	0	31	6.23	−42	4	0
		L						17	5.87	−44	0	2										
Thalamus		R	32	8.11	22	−30	−2						31	6.84	22	−30	−2	16	6.29	20	−28	−2
		L																13	5.53	−16	−26	0
Hippocampus		R	32	7.24	20	−32	−2						31	6.46	24	−28	−6	55	5.18	22	−30	−4^*^
		L						93	5.44	−24	−26	−8						65	4.73	−20	−30	−4^*^
Parahippocampal Gyrus	27	R	12	5.52	20	−34	−4															
Fusiform Gyrus	37	R	57	6.83	26	−52	−12	19	6.05	28	−52	−14										
	20	R																14	7.46	32	−38	−24

* *SVC FWE-corrected on cluster level p < 0.05*.

#### Direct comparisons of MEM and PLAC groups

##### Extinction

**Figure 3:**
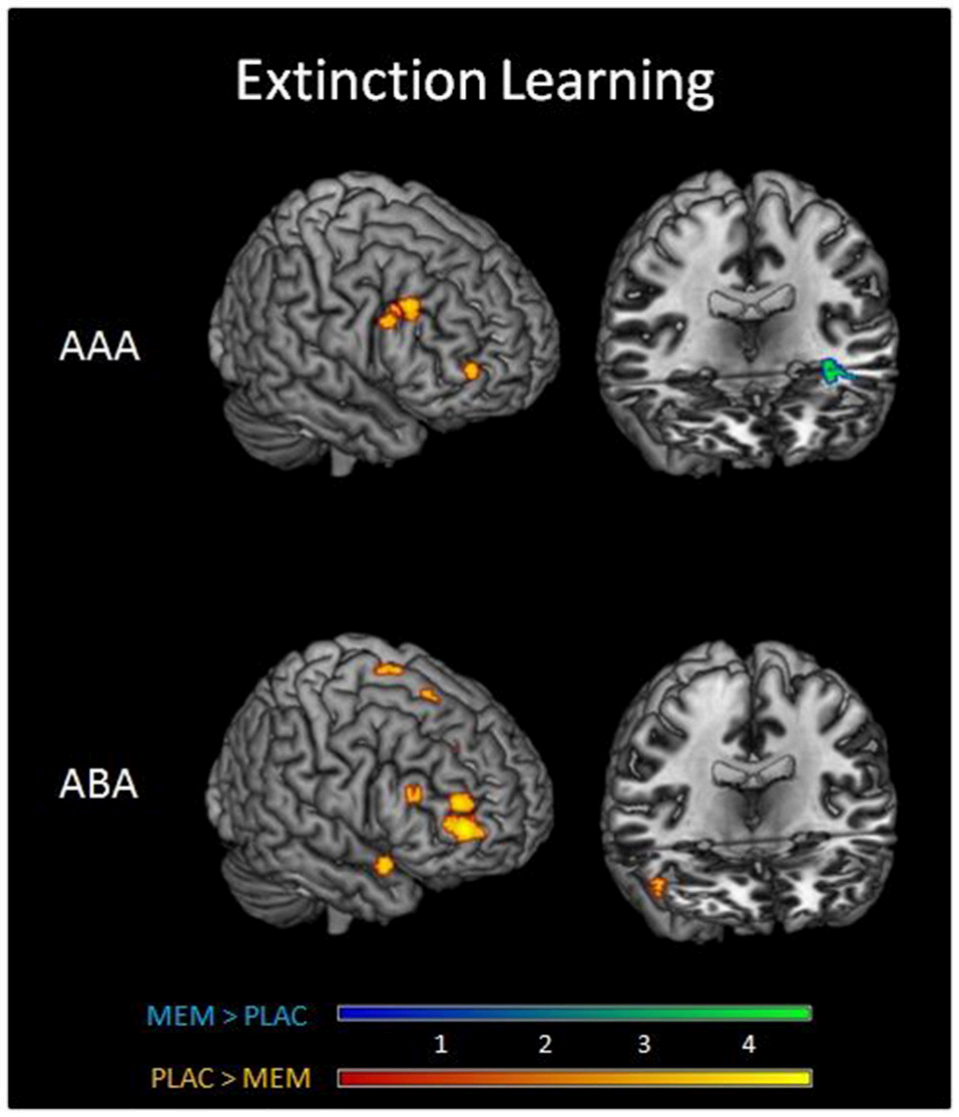
**Imaging results in Extinction**. Overlays of activation patterns of the PLAC (yellow-red) and MEM group (blue-green) during AAA (top) and ABA (bottom) extinction learning. The direct comparison of both groups showed substantially reduced activation in the MEM group compared to PLAC during AAA and ABA extinction in right prefrontal cortex. MEM participants, however, showed higher activation in left hippocampus exclusively in AAA extinction (two sample *t*-tests *p* < 0.001 uncorrected, minimum cluster size *k* = 10, with age and gender as nuisance variables).

A two-sample *t*-test showed substantially reduced activation in the MEM group compared to PLAC during AAA and ABA extinction in right dlPFC (BA9) and OFC (BA10) as well as in STG (BA38). In contrast, MEM participants showed higher activation in left hippocampus exclusively in AAA extinction (see Table 3 and Figure 3).

**Table 3:** **Two-sample tests (age and gender as nuisance variables)—activated regions (MNI coordinates) in MEM and PLAC during extinction learning, peak cluster *p* < 0.001 uncorrected, *k* = 10**.

**Brain region**	**BA**	**Hem**	**AAA CC**										**ABA CC**									
			**MEM > PLAC**					**PLAC > MEM**					**MEM > PLAC**					**PLAC > MEM**				
			**voxel**	***t*-value**	***x***	***y***	***z***	**voxel**	***t*-value**	***x***	***y***	***z***	**voxel**	***t*-value**	***x***	***y***	***z***	**voxel**	***t*-value**	***x***	***y***	***z***
dlPFC		R																44	4.18	38	50	20
		R																130	4.58	38	54	4
		R						41	3.98	52	26	36						15	3.82	52	32	24
	9	R						13	3.56	56	18	28						10	3.64	6	30	38
OFC	10	R						17	3.94	34	54	4										
STG	38	R	12	3.62	−32	16	−24											21	3.98	52	16	−14
Hippocampus		L	50	3.95	−38	−20	−12															

##### Recall

The two-sample *t*-test did not yield any significant activation differences between the groups in AAA and ABA recall.

### Pharmacological effects modulated by BMI

The unexpected enhancement of extinction learning performance and hippocampal activation present in the MEM group suggests that the standardized dose of memantine that we administered partially displayed neuroenhancing properties. On the other hand, the observed activation decrease in prefrontal regions demonstrates an effect expected from an NMDA antagonist. To reconcile these findings, we explored to what extent the effect of the drug depended on interindividual differences in its bioavailability, which might or might not support neuroenhancing effects. Bioavailability might be related to participants' body weight, or more specifically, their body fat. Since memantine possesses lipophilic pharmacological characteristics (Henkel et al., 1982), we used the BMI—instead of the body weight alone—as a marker that provides a better indication for the body stature and takes relative body fat content into consideration (WHO, 1998).

**Figure 4:**
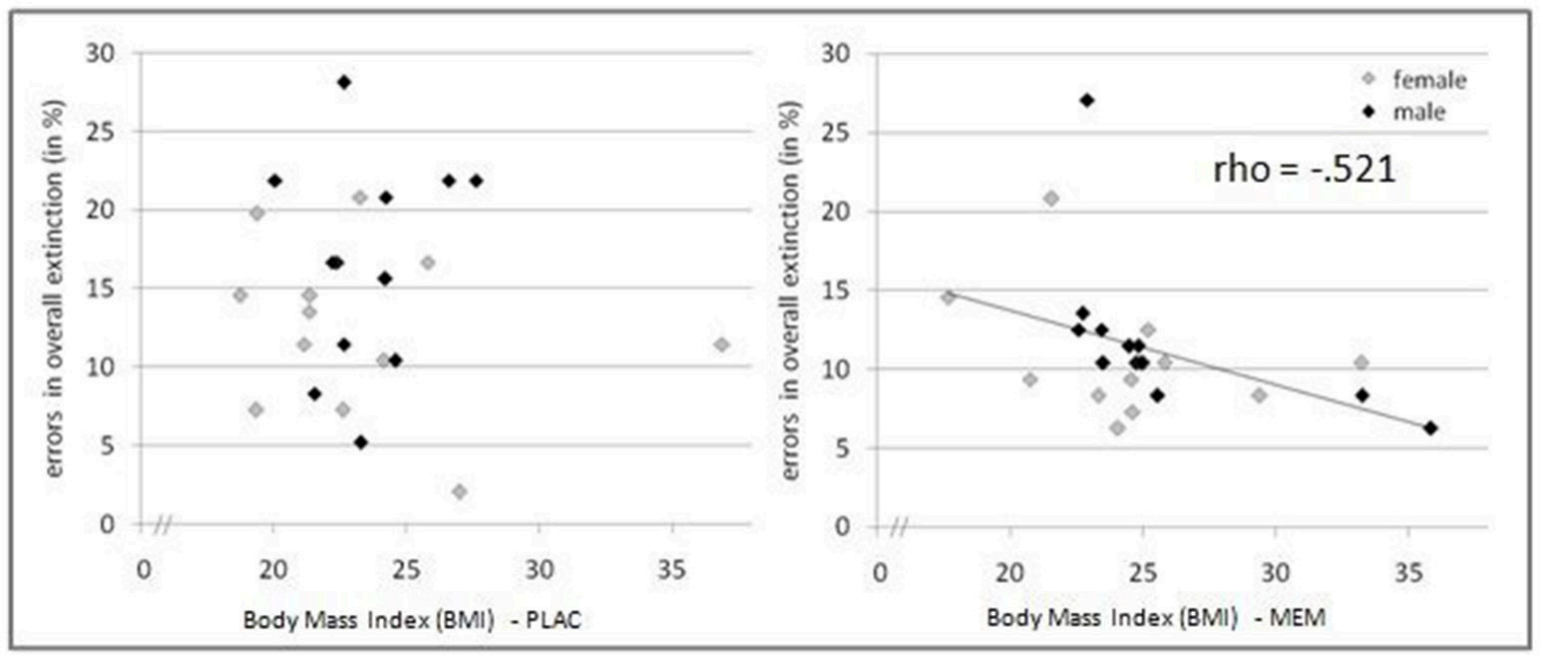
**Pharmacological effects modulated by BMI**. Relation between BMI and extinction errors for PLAC (left) and MEM participants (right). Only in the MEM group a significant negative correlation was observed (*rho* = −0.521, *p* < 0.01, one-tailed): Higher BMI values were associated with fewer extinction errors.

The mean BMI of the MEM and PLAC groups did not differ significantly: *t*_(46)_ = 1.545, *p* = 0.129; PLAC 25.17 ± 0.83, MEM 23.45 ± 0.76. We calculated correlations of acquisition and extinction learning performance with the participants' BMI. As expected, in the acquisition phase performed prior to drug administration we observed no significant correlation between BMI and number of errors for both groups (MEM group *rho* = 0.244, *p* = 0.125, one-tailed; PLAC group *rho* = 0.097, *p* = 0.326, one-tailed). Interestingly, during extinction learning after drug administration MEM participants' BMI was negatively correlated with their number of extinction errors (*rho* = −0.521, *p* < 0.01, one-tailed). However, again no significant correlation was found for PLAC participants (*rho* = 0.031, *p* = 0.443, one-tailed). Thus, only in participants who had received memantine, the correlation of BMI and extinction learning performance indicated that higher BMI values were associated with fewer extinction errors (see Figure 4).

**Figure 5:**
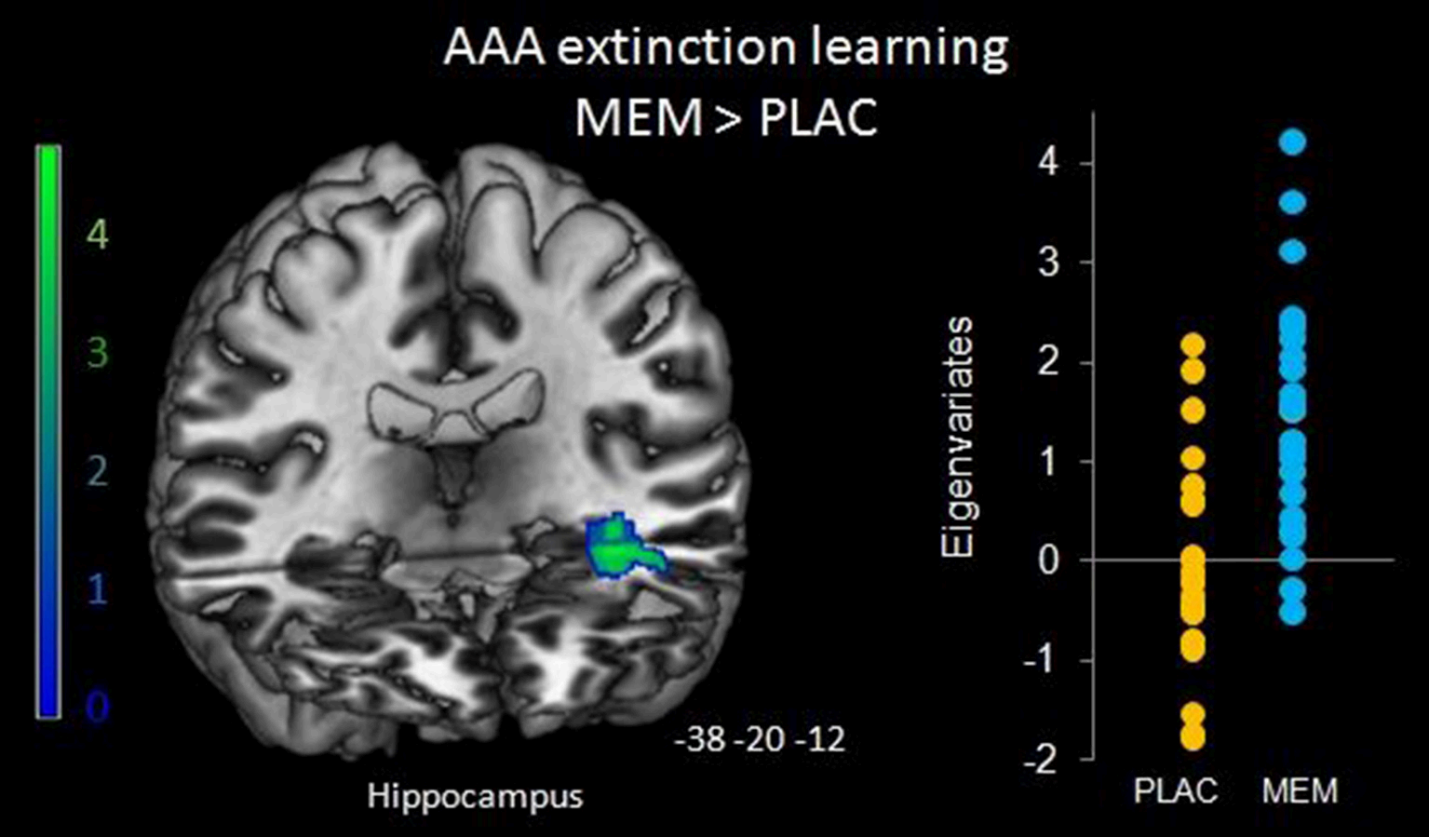
**Hippocampal activation during AAA extinction covarying with BMI and extinction errors**. Higher left hippocampal activation in the MEM group (compared to PLAC) that covaried with BMI and extinction errors during AAA extinction learning (SPM analyses of MEM > PLAC thresholded at *p* < 0.05 FWE-corrected on cluster level, minimum cluster size *k* = 20, with BMI and extinction errors as covariates of interest and age and gender as nuisance variables). The bar graph represents the extracted eigenvariates of the hippocampus cluster separately for PLAC and MEM participants.

To determine the effect of the BMI on brain activation levels in extinction learning we used a flexible factorial SPM design with factors BMI and extinction errors as covariates of interest and age and gender as nuisance variables. Based on the negative correlation between BMI and extinction errors in MEM participants, we hypothesized that the BMI was positively associated with brain activation, whereas a negative association was assumed between brain activation and the number of extinction errors. Therefore, BMI entered the analyses as a positive and errors as a negative covariate. Separate analyses were performed for extinction learning in AAA and ABA conditions. During extinction learning in the AAA condition, higher BOLD activation in the left-hemispheric middle hippocampus in MEM compared to PLAC participants correlated with BMI and extinction errors (MNI coordinates *x* = −38 *y* = −20 *z* = −12) (see Figure 5). This finding suggests that the higher hippocampus activation observed already in the two-sample *t*-tests (see Table 3 and Figure 3), predominantly resulted from the contribution of participants with higher BMI. In contrast, in PLAC compared to MEM participants no brain activation covaried with BMI and error rate. In the ABA condition, no differences between the groups were observed. Also, for extinction recall no significant group differences can be reported, neither in the AAA nor in the ABA condition.

## Discussion

In this study we investigated the involvement of NMDAR in context-related extinction learning as well as their role for renewal. Results show that a single dose of the NMDAR antagonist memantine, administered prior to the extinction session, modulates behavioral performance as well as neural correlates of extinction learning:

- NMDAR antagonism enhances extinction learning particularly for familiar context-cue compounds.
- Effects of memantine upon extinction learning performance are related to participants' body mass index.
- Enhancement in AAA extinction learning is associated with higher hippocampus activation.
- The NMDAR antagonist reduces prefrontal activation during extinction learning.
- NMDAR antagonism does not affect renewal.

### NMDAR antagonism enhances extinction learning particularly for familiar context-cue compounds

Against our initial hypothesis, we observed better overall extinction learning in MEM participants compared to PLAC, a result which suggests a higher potential for behavioral flexibility in MEM participants. In particular, this observed enhancement was based on a better performance in AAA extinction: Here, the MEM group made fewer errors in linking the familiar context-cue compound to a changed outcome. This superior performance was reflected in their faster AAA extinction learning as demonstrated by an analysis of the groups' learning curves. However, no differences were observed in ABA trials. A potential reason may be that the processing requirements in these two conditions differ: In AAA trials, the familiar context-cue compound merely has to be associated with a changed outcome, whereas in ABA trials a novel context-cue compound is presented, in addition to the change in outcome. Thus, ABA trials presumably pose an additional learning challenge, which, of note, was previously found impaired by a DA-antagonist (Lissek et al., 2015b). It is therefore conceivable that the observed effects of the NMDAR antagonist were primarily based on its ability to alter the association of a familiar context-cue compound with an outcome. In contrast, the processing of an additionally changed element, i.e., a novel context, as required in the ABA condition, apparently was not influenced by NMDAR antagonism.

Our findings are in contrast to results from animal studies which reported impairments in extinction learning following NMDAR blockade (Falls et al., 1992; Davis et al., 2003; Lissek and Güntürkün, 2003; Burgos-Robles et al., 2007; Sotres-Bayon et al., 2009). However, the capability of memantine to act as a neuroenhancer in humans, which may improve cognitive functions in healthy individuals (for review see Repantis et al., 2010), could be a conceivable explanation for these discrepancies. Even though due to divergent acute effects after a single dose of memantine, Repantis et al. (2010) were not able to adequately estimate memantine's potential for neuroenhancement, an enhancing effect of memantine cannot be excluded. More research is necessary to evaluate potential enhancing effects of a single dose of memantine upon healthy humans.

### Effects of memantine upon extinction learning performance are related to participants' body mass index

In the present study, in which a standardized single dose of memantine was administered, participants' BMI influenced the effects of the drug: Higher BMI values were associated with fewer extinction errors. Since all participants received an identical dose of 30 mg memantine, it is conceivable that those with a higher BMI might have established a lower metabolically effective dose, whereas those with a lower BMI might have established a higher metabolically effective dose. In fact, dose-dependent effects are a plausible explanation for our findings in view of memantine's lipophilic pharmacological characteristics (Lipophilicity is expressed as *log P* = 3.28; Henkel et al., 1982), whereby a higher amount of body fat is associated with a slower metabolization of memantine. Thus, the administration of the same dose presumably leads to a lower plasma concentration of memantine at the time of testing in participants with high BMI (compared to low BMI) which in turn may have caused enhanced performance. Unfortunately, however, our study did not provide adequate conditions to validate this assumption. Therefore, our attempt at an explanation for this a posteriori result must be considered speculative. To disentangle potential dose-dependent effects influenced by participants' BMI, future studies might analyze potential differences in peak plasma concentrations of participants with high vs. low BMI.

Our results correspond to previous findings on NMDA antagonism in humans, with dose-dependent learning and memory effects observed for the non-competitive NMDAR antagonist ketamine, revealing impaired recall at high doses (Newcomer and Krystal, 2001). Likewise, animal studies in rats highlighted dose-dependent effects in extinction learning: An intra-amygdala injection of the NMDAR antagonist AP5 caused dose-dependent impairments in the expression of extinction (Falls et al., 1992). Moreover, Baker and Azorlosa (1996) replicated these results by blocking NMDAR with MK-801, and reported better extinction learning performance under low dose, whereas rats receiving a high dose of MK-801 behaved similar to the controls which had not experienced extinction trials. In accordance, the infusion of NMDAR agonist D-cycloserine (DCS) into the basolateral amygdala revealed greater enhancing effects in extinction learning for higher doses (Walker et al., 2002; Ledgerwood et al., 2003).

However, future studies need to examine the effects of different dosages upon individuals in a within-subject design. Up to now, only a few studies focused on the effects of memantine upon learning and memory in healthy human participants. Of note, most of these human studies examined the effects of memantine only in men, and did not take participants' BMI into account. Those few studies that involved men and women did not consider sex differences or BMI distributions (see e.g., Korostenskaja et al., 2007). However, the results of the present study indicate that using a standardized dose of memantine irrespective of BMI may mask effects of NMDA antagonism upon learning. Thus, the present study results may provide a first explanation for divergent effects of memantine reported in the literature. Accordingly, to reconcile previous findings, further research is highly recommended to disentangle possible dose-dependent effects of memantine.

### Enhancement in AAA extinction learning is associated with higher hippocampus activation

To explore a potential modulator of the enhanced learning performance, we analyzed participants' hippocampal activation in more detail. While both MEM and PLAC groups recruited hippocampal regions during ABA and AAA extinction learning, as well as during ABA recall, a substantially higher activation in hippocampus and parahippocampal gyrus was only present in the MEM group during AAA extinction.

This hippocampal activation showed a significant association with BMI and extinction errors exclusively for MEM compared to PLAC. These results suggest that uncoupling a familiar context-cue-compound from its associated outcome and connecting it with a different outcome was facilitated by enhanced NMDAergic processing in hippocampus in the MEM group, which in turn led to their lower error rates in AAA extinction.

In contrast, extinction in the ABA condition recruited hippocampus to a similar degree in both the MEM and PLAC groups, demonstrating that the presentation of novel context information did not evoke a higher hippocampal response in MEM than in PLAC participants. As shown in the present study and several other previous studies (Lissek et al., 2015a,b, 2016), hippocampal activation during contextual extinction learning is not unique to extinction in a novel context, but also occurs during processing of extinction in an identical context. Thus, it is conceivable that the MEM group's higher hippocampal activation during AAA extinction was not driven predominantly by novelty or context properties, but may have supported the adaptation of their response to a familiar context-cue compound and helped incorporating the altered outcome by re-evaluating the contextual association potentially established during acquisition. In summary, this result suggests that hippocampal NMDAR are involved in behavioral flexibility required in extinction learning.

Taken together, our findings suggest that memantine has a dose-associated mode of action in hippocampal regions, whereas NMDAR activity in prefrontal areas is largely blocked irrespective of the metabolically effective dose. A potential explanation for dose-dependent effects of memantine upon hippocampus comes from previous findings which indicate that hippocampal NMDAR are dynamically organized, meaning that unblocked receptors can be moved into preexisting synapses (Tovar and Westbrook, 2002). According to the researchers' findings, 65% of the NMDAR in hippocampus are mobile. This special characteristic may support dose-dependent effects of memantine: One can assume that in cases of a low dose more NMDAR will remain unblocked and therefore free to move into preexisting synapses. This dynamic organization can lead to increasing efficiency in input processing. Accordingly, a high dose of memantine will block most of the NMDAR, depleting the reservoir of mobile receptors. This may lead to a complete NMDAR blockade, resulting in impaired synaptical processing. In summary, such an interpretation may help to explain conflicting results of hippocampal NMDAR blockade upon learning and memory processes found in previous studies (for review see Parsons et al., 2007).

### The NMDAR antagonist reduces prefrontal activation during extinction learning

In parallel to improved extinction learning, the MEM group showed decreased BOLD activation during extinction learning particularly in prefrontal (dlPFC, OFC) and temporal (STG) regions. Our results are in line with previous findings, which demonstrated reduced activation in PFC extending to the anterior cingulate cortex (ACC) without behavioral impairment after memantine administration (Van Wageningen et al., 2009b). Another study using the NMDAR antagonist ketamine also showed no changes in performance together with alterations in brain networks subserving associated cognitive processes (Deakin et al., 2008).

In summary, recent fMRI studies using NMDAR antagonists reported reduced activation in prefrontal regions, which can also be associated with decreased dlPFC network connectivity (Driesen et al., 2013). However, at lower doses of the NMDA antagonist, prefrontal deactivation does not necessarily affect behavioral performance, indicating that alterations in neuronal responses could occur prior to a behavioral deviation (Van Wageningen et al., 2009a)—an observation which corresponds to our findings of reduced prefrontal activation without a behavioral impairment.

Thus in our study, the observed modulation of PFC activity apparently did not have any effect upon extinction learning, whose enhancement was most probably supported by the increased hippocampal activation. This pattern corresponds to that found by Deakin et al. (2008) who after ketamine administration also observed an increased BOLD response in hippocampus, in parallel to a decrease in OFC—providing converging evidence which demonstrates that the impact of NMDAR antagonists is not necessarily consistent across all affected brain regions.

In addition to a pattern of reduced prefrontal activation, one-sample tests also yielded decreased amygdalar activation in MEM participants. The amygdala appears to be necessary for updating representations of value, not only in fear extinction, but also in reinforcer devaluation in order to coordinate physiological, behavioral, and cognitive responses in an affective/emotional context (Morrison and Salzman, 2010). Previous studies highlighted the essential activation of amygdalar NMDAR for (fear) extinction (for review see Davis et al., 2003): While the blockade of NMDAR in rats impaired extinction learning (Falls et al., 1992; Santini et al., 2001; Burgos-Robles et al., 2007), their stimulation enhanced extinction learning processes (Walker et al., 2002; Ledgerwood et al., 2003). In contrast, in our study the inhibition of amydalar NMDAR activation associated with the administration of memantine did not affect general learning performance, suggesting that amygdalar processing has only a minor role in associative extinction learning as used in this study.

Our findings support a notion of amygdala NMDAR activation being predominantly relevant for fear extinction learning (Davis, 2002) as well as reward-based decision making processes (Morrison and Salzman, 2010) rather than for non-fear-related extinction tasks.

### NMDAR antagonism does not affect renewal

No differences between the groups were observed in ABA renewal: In both groups, a similar proportion of participants showed renewal, and the percentage of renewal effect responses was similar in both groups. Thus, the recall of previously established associations was not affected by blocking NMDAR, a finding which corresponds to the results of previous studies using other non-competitive NMDA antagonists: Hetem et al. (2000) showed that ketamine did not affect the retrieval of previously learned words, a finding which was also replicated by Rowland et al. (2005), highlighting that ketamine did not influence retrieval of verbal information. Likewise, ketamine did not affect retrieval of a previously learned rule (i.e., Wisconsin Card Sorting Test) (Krystal et al., 2000). Thus, converging evidence suggests that across different tasks NMDAR blockade does not affect the recall of previously learned associations.

### Comments on task design and observed renewal rates

Despite obvious differences in processing requirements, previous studies highlighted the parallels between context-related extinction learning with and without a fear component: In both, fear (for review see Bouton, 2002, 2004) and non-fear related extinction (Rosas and Callejas-Aguilera, 2006; Rosas et al., 2006; Ungör and Lachnit, 2006; Üngör and Lachnit, 2008; Lucke et al., 2013, 2014), the renewal effect has been demonstrated. Moreover, recent imaging/lesion studies emphasized involvement of similar brain regions in both types of extinction learning, in animals as well as in humans. Despite prominent amygdalar activation during fear conditioning (for review see Sehlmeyer et al., 2009), particularly hippocampal and prefrontal activation play a crucial role in encoding and integrating context information during associative extinction learning (Lissek et al., 2013) as well as during fear extinction respectively recall (Corcoran and Maren, 2001; Corcoran et al., 2005; Kalisch et al., 2006; Milad et al., 2007). Based on these behavioral as well as neural parallels, extinction studies with and without a fear component appear largely comparable. Moreover, extinction studies without a fear component can extend our understanding of the common neural mechanisms that underlie all extinction learning.

In the present study, we observed rather low renewal rates, compared to previous studies using the same or a similar predictive learning task (Lissek et al., 2013, 2015a,b; Kinner et al., 2016). The interval length between acquisition and extinction as well as between extinction and recall has been shown to play a role for renewal in general, with longer intervals between each of these phases resulting in lower renewal rates—an effect that has been observed in various studies on human fear extinction (Huff et al., 2009; Merz et al., 2016) and is supported by our previous findings with the predictive learning task. Therefore, conceivably, the low renewal level is related to the 24 h interval between acquisition and extinction phases that was introduced to prevent effects of the NMDA antagonist upon consolidation of acquisition. The performance of extinction and recall phases back-to-back, in contrast, should not have the potential to reduce renewal. In previous studies with the predictive learning task, the same interval between extinction and recall was used and yielded substantially higher renewal rates (Lissek et al., 2013, 2015a,b).

Moreover, extinction and recall phases differ with regard to the feedback provided—during extinction the feedback states the correct response, while during recall no more feedback is given. Therefore, the recall phase does not constitute an extension of the extinction phase, since it requires recall of previously acquired associations without ongoing feedback or reinforcement.

## Conclusion

To the best of our knowledge, this study is the first to investigate the effects of NMDAR blockade upon brain activation associated with extinction learning and renewal in healthy human participants. Our findings deliver evidence for the involvement of the human NMDA system in changing established cue-outcome associations during extinction learning: On the one hand, the NMDA antagonist caused a decrease in activation during extinction and recall, especially in prefrontal (dlPFC, OFC) and temporal (STG) brain regions. On the other hand, the NMDA antagonist enhanced performance in extinction learning in parallel to higher hippocampal activation which was correlated with participants' BMI and error rate. Hippocampal activation may contribute to more efficient uncoupling of associations between an established context-cue-compound and an outcome. In contrast, NMDAR blockade did not affect recall of previously established associations. In summary, the NMDAergic system appears to support behavioral flexibility in extinction learning, which seems to be dose-related: While an NMDAR blockade with low efficiency may support targeted processing, a blockade with high efficiency may cause impairment. Further studies are necessary to disentangle possible dose-dependent acute effects of a single dose of memantine on extinction learning.

## Ethics statement

This study was carried out in accordance with the recommendations of the Ruhr University Bochum with written informed consent from all subjects. All subjects gave written informed consent in accordance with the Declaration of Helsinki. The protocol was approved by the local ethics board of the Ruhr University Bochum.

## Author contributions

MT and SL: Developed study conception and design. SL and AG: Principal investigators, interpretation of data, wrote manuscript. AG and BG: Acquired data. AG: Performed data analyses. AG and SH: Performed statistics. SH, BG and MT: Made critical revisions. All authors contributed to and have approved the final manuscript.

## Funding

This work was supported by a grant from the Deutsche Forschungsgemeinschaft (FOR 1581 Extinction Learning) and the DGUV (FP 365).

### Conflict of interest statement

The authors declare that the research was conducted in the absence of any commercial or financial relationships that could be construed as a potential conflict of interest.
